# 4f-Metal Clusters Exhibiting Slow Relaxation of Magnetization: A {Dy_7_} Complex with An Hourglass-like Metal Topology

**DOI:** 10.3390/molecules25092191

**Published:** 2020-05-07

**Authors:** Konstantinos N. Pantelis, Panagiota S. Perlepe, Spyridon Grammatikopoulos, Christos Lampropoulos, Jinkui Tang, Theocharis C. Stamatatos

**Affiliations:** 1 Chemistry Department, University of Patras, 265 04 Patras, Greece; kostaspantelis95@gmail.com (K.N.P.); pennyperlepes@gmail.com (P.S.P.); spiridongramma@upatras.gr (S.G.); 2 Department of Chemistry, University of North Florida, 1 UNF Dr., Jacksonville, FL 32224, USA; christosl1981@gmail.com; 3 State Key Laboratory of Rare Earth Resource Utilization, Changchun Institute of Applied Chemistry, Chinese Academy of Sciences, Changchun 130022, China; tang@ciac.ac.cn

**Keywords:** polynuclear metal complexes, dysprosium(III), Schiff base ligands, *N*-naphthalidene-2-amino-5-chlorobenzoic acid, single-crystal X-ray crystallography, single-molecule magnets

## Abstract

The reaction between Dy(NO_3_)_3_∙6H_2_O and the bulky Schiff base ligand, *N*-naphthalidene-2-amino-5-chlorobenzoic acid (nacbH_2_), in the presence of the organic base NEt_3_ has led to crystallization and structural, spectroscopic and magnetic characterization of a new heptanuclear [Dy_7_(OH)_6_(OMe)_2_(NO_3_)_1.5_(nacb)_2_(nacbH)_6_(MeOH)(H_2_O)_2_](NO_3_)_1.5_ (**1**) compound in ~40% yield. Complex **1** has a unique hourglass-like metal topology, among all previously reported {Dy_7_} clusters, comprising two distorted {Dy_4_(μ_3_-OH)_3_(μ_3_-OMe)}^8+^ cubanes that share a common metal vertex (Dy2). Peripheral ligation about the metal core is provided by the carboxylate groups of four η^1^:η^1^:η^1^:μ single-deprotonated nacbH^−^ and two η^1^:η^1^:η^2^:η^1^:μ_3_ fully-deprotonated nacb^2−^ ligands. Complex **1** is the first structurally characterized 4f-metal complex bearing the chelating/bridging ligand nacbH_2_ at any protonation level. Magnetic susceptibility studies revealed that **1** exhibits slow relaxation of magnetization at a zero external dc field, albeit with a small energy barrier of ~5 K for the magnetization reversal, most likely due to the very fast quantum-tunneling process. The combined results are a promising start to further explore the reactivity of nacbH_2_ upon all lanthanide ions and the systematic use of this chelate ligand as a route to new 4f-metal cluster compounds with beautiful structures and interesting magnetic dynamics.

## 1. Introduction

The recent interest in 4f-metal chemistry has leaned towards the fields of molecular nanomagnetism, spintronics and quantum computation [1]. This is primarily due to the ability of several lanthanide complexes to act as single-ion/molecule magnets (SIMs/SMMs) under an appropriate crystal field [2,3,4]. The large number of unpaired electrons, in combination with the significant magnetic anisotropy originating from the unquenched spin-orbit coupling and the ligand field effects, of some lanthanide ions, such as Dy^III^ and Tb^III^, render them promising candidates for SIM/SMM behaviors [5,6,7,8]. The latter are molecular, 0-D compounds that exhibit slow relaxation of their magnetization in the absence of an external magnetic field [9], as evidenced by the appearance of frequency-dependent out-of-phase (*χ*_Μ_′′) signals below a blocking temperature (*T*_B_) and hysteresis loops, the diagnostic property of a magnet [10]. To this end, the optimization of the lanthanide’s coordination polyhedron appears as a prerequisite for the synthesis of efficient SIMs with large energy barriers (*U*_eff_) and high *T*_B_ [11]; indeed, a recently reported organometallic dysprosium metallocene displayed magnetic hysteresis above liquid-nitrogen temperatures and a *U*_eff_ barrier of 1541 cm^−1^ [12].

In contrast to mononuclear SIMs, the polynuclear 4f-metal clusters usually exhibit weak SMM behaviors due to the fast-tunneling rates, which is a consequence of the different lanthanide coordination environments with distorted, low-symmetry geometries and the random orientation of the individual anisotropy axes [13]. However, this drawback is compensated by the aesthetically beautiful structures that these nanoscale molecular compounds frequently possess [14]. These structural motifs often resemble, at the molecular level, the repeating units and properties of some important nanoscale materials, such as brucites, perovskites and honeycombs, to name a few. The aggregation process in 4f-metal cluster chemistry is mostly based on the metal-assisted hydrolysis reactions in the presence of organic chelating/bridging ligands, which assist towards the stabilization of the internal {Ln_x_O_y_(OH)_z_} metal cores and the crystallization of the resulting molecular species [15]. Hence, it becomes apparent that the choice of the organic chelate ligand is of paramount importance in the quest for high-nuclearity 4f-metal complexes and SMMs.

**Scheme 1:**
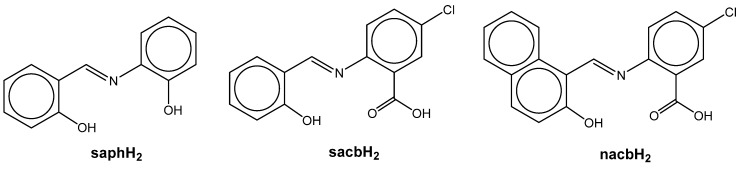
Structural formulae and abbreviations of the Schiff base ligands discussed in the text.

Our group has had a longstanding interest in the use of Schiff base ligands that resemble the scaffold of the well-known *N*-salicylidene-o-aminophenol (saphH_2_, Scheme 1) chelate towards the synthesis of nanosized 3d- and 4f-metal compounds with potentially interesting magnetic and optical properties [16,17]. The first member of this family of chelates has been the *N*-salicylidene-2-amino-5-chlorobenzoic acid (sacbH_2_, Scheme 1), a tetradentate chelating and bridging ligand that has proved its ability to stabilize high-nuclearity 3d-metal clusters [18] and polymers [19] with unprecedented structures but with rather trivial magnetic properties. However, when sacbH_2_ was used in Dy^III^ chemistry, a small in nuclearity {Dy_2_} compound was isolated, but its magnetic characterization unveiled an interesting SMM behavior with an energy barrier of 109 K and out-of-phase peaks of signals below ~25 K [20]. Based on these findings, we have decided to turn our attention to the chemically and electronically similar ligand *N*-naphthalidene-2-amino-5-chlorobenzoic acid (nacbH_2_, Scheme 1), which is sterically though more rigid, and its solubility in organic solvents differs significantly from that of sacbH_2_. These combined factors were the main reasons for the isolation and structural characterization of a series of totally different 3d-metal/nacb^2−^ clusters [21,22] when compared to the obtained results from similar systems with sacbH_2_ chelate.

Given the absence of any previous use of nacbH_2_ in 4f-metal chemistry, we herein report our first results from the study of the tertiary Dy^III^/NO_3_^−^/nacbH_2_ system, which has led us to the crystallization, spectroscopic and magnetic characterization of a new [Dy_7_(OH)_6_(OMe)_2_(NO_3_)_1.5_(nacb)_2_(nacbH)_6_(MeOH)(H_2_O)_2_](NO_3_)_1.5_ (**1**) cluster with a unique hourglass-like metal topology and slow relaxation of magnetization. It is worth noting that the same reaction system, but with sacbH_2_ in place of nacbH_2_, has yielded the dinuclear compound [Dy_2_(NO_3_)_4_(sacbH)_2_(H_2_O)_2_(MeCN)_2_] with different structural and magnetic properties [20].

## 2. Results and Discussion

### 2.1. Synthetic Comments

At first glance, the tertiary Dy^III^/NO_3_^−^/nacbH_2_ reaction system appears as a simple synthetic scheme to employ for the preparation of high-nuclearity 4f-metal clusters. However, this route allows nacbH_2_ to unveil (under the deprotonated forms, i.e., nacbH^−^ and/or nacb^2−^) its bridging/chelating affinity to the lanthanide metal centers, free of any ancillary bridging anions, such as carboxylates, β-diketonates or pseudohalides, which are frequently used in more complicated reactions schemes [23]. The NO_3_^−^ ions can—in principle—play a dual role; they can act as bidentate chelating groups (through their O-donor atoms) to fulfill the large coordination numbers of the oxophilic 4f-metal ions, and they can also act as counterions to stabilize cationic cluster compounds in solution and, subsequently, in the solid-state. In such a reaction scheme, the presence of an external organic base, such as NEt_3_, is an important synthetic parameter; a relatively weak base can behave as a proton acceptor to facilitate the complete deprotonation of the organic chelate, thus unfolding all the coordination capacity of the ligand’s donor atoms, and promote further the metal-assisted deprotonation of H_2_O (from starting materials and solvents) to O^2−^ and/or OH^−^ groups, which are usually holding the metal centers together in the internal cluster cores [24]. A strong base, such as R_4_NOH (R = Me, Et, Bu), could also play a similar role, but their use is often accompanied by the formation of amorphous lanthanide oxides or hydroxides, which are detrimental to the crystallization of a high-nuclearity compound. Therefore, the 1:1:3 reaction between Dy(NO_3_)_3_∙5H_2_O, nacbH_2_ and NEt_3_, in a solvent mixture comprising MeOH and MeCN, has led to the heptanuclear cluster compound [Dy_7_(OH)_6_(OMe)_2_(NO_3_)_1.5_(nacb)_2_(nacbH)_6_(MeOH)(H_2_O)_2_](NO_3_)_1.5_ (**1**) as yellow needle-like crystals of 40% isolated yield. The general formation of **1** is summarized by the following stoichiometric Equation (1).

7 Dy(NO_3_)_3_∙5H_2_O + 8 nacbH_2_ + 3 MeOH + 18 NEt_3_ → [Dy_7_(OH)_6_(OMe)_2_(NO_3_)_1.5_(nacb)_2_(nacbH)_6_(MeOH)(H_2_O)_2_](NO_3_)_1.5_ +
18 (Et_3_NH)(NO_3_) + 27 H_2_O (1)

The choice of the organic solvate media was proved to be a decisive factor for the growth of single crystals of **1**∙5MeOH∙MeCN suitable for X-ray diffraction studies. When only MeOH was used as the reaction solvent, a yellow crystalline solid was isolated and identified as **1**, on the basis of IR spectroscopy and elemental (C, H and N) analyses. In contrast, when MeCN was solely used as the reaction solvent, orange-colored amorphous precipitates were formed that we were unable to characterize and derive any accurate conclusions. Reactions in different solvent mixtures did not afford any additional crystalline products. Undoubtedly, MeOH is a key reagent for the synthesis and crystallization of **1**; in addition to all the aforementioned characteristics, MeOH appears as the only supplier of bridging MeO^−^ groups within the structure of **1** (vide infra).

### 2.2. Description of Structure

**Figure 1:**
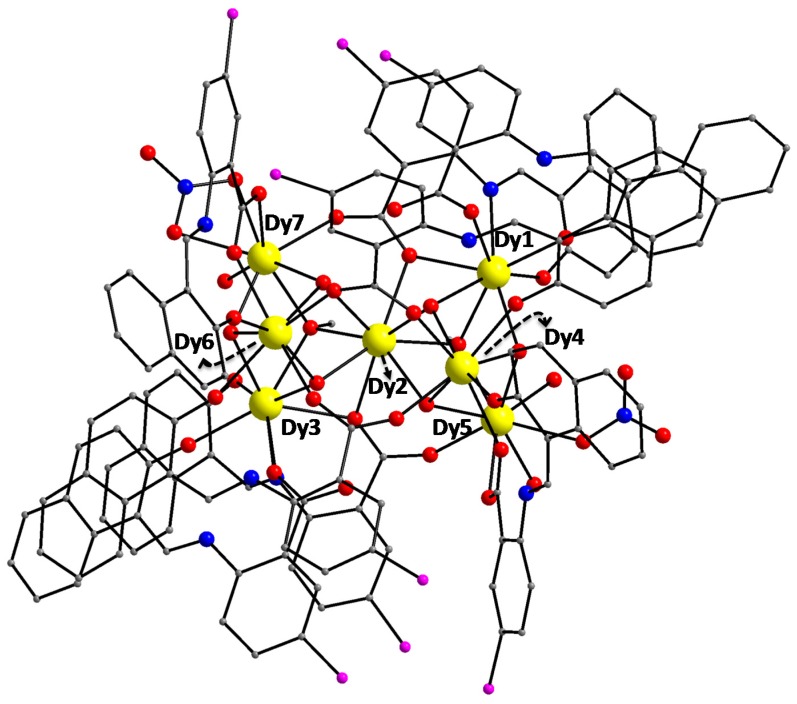
Partially labeled representation of the cation of complex **1**. Color scheme: Dy^III^, yellow; Cl, cyan; O, red; N, blue and C, gray. H atoms are omitted for clarity.

A partially labeled representation of the cation of complex **1** is shown in Figure 1. The cluster cation [Dy_7_(OH)_6_(OMe)_2_(NO_3_)_1.5_(nacb)_2_(nacbH)_6_(MeOH)(H_2_O)_2_]^1.5+^ is counterbalanced by three NO_3_^−^ counterions of 50% occupancy each in the crystal lattice. The NO_3_^−^ counterions, together with the lattice solvate molecules (MeOH and MeCN), occupy the voids between the {Dy_7_} clusters in the crystal lattice (Figure S1). Selected interatomic distances and angles of **1** are listed in Table 1. Bond valence sum (BVS) calculations for the inorganic μ_3_-bridging O-atoms gave values of 1.30 (for O2), 1.15 (for O4), 1.29 (for O6), 1.01 (for O8), 1.07 (for O14) and 1.18 (for O16) in excellent agreement with their assignment as OH^−^ groups. Furthermore, BVS calculations for the terminally bound O25 and O39 atoms gave the same value of 0.39, assigned to coordinated H_2_O molecules. Oxygen BVS values in the ~1.7–2.0, ~1.0–1.3 and ~0.2–0.4 ranges are indicative of non-, single- and double-protonation, respectively [25].

**Figure 2:**
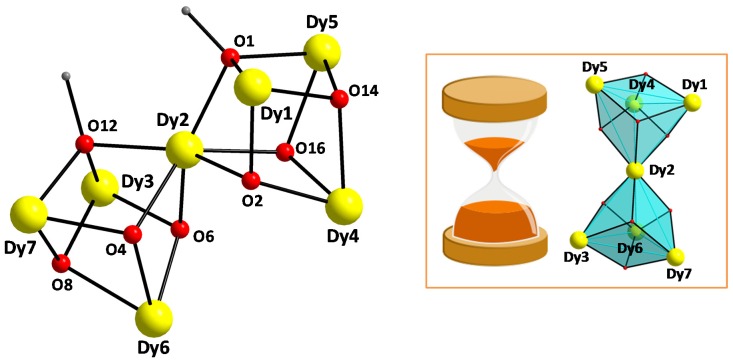
(left) The metal vertex-sharing, dicubane {Dy_7_(μ_3_-OH)_6_(μ_3_-OMe)_2_}^13+^ core of **1** and (right, enclosed in the box) its hourglass-like metal topology. Color scheme as in Figure 1.

**Figure 3:**
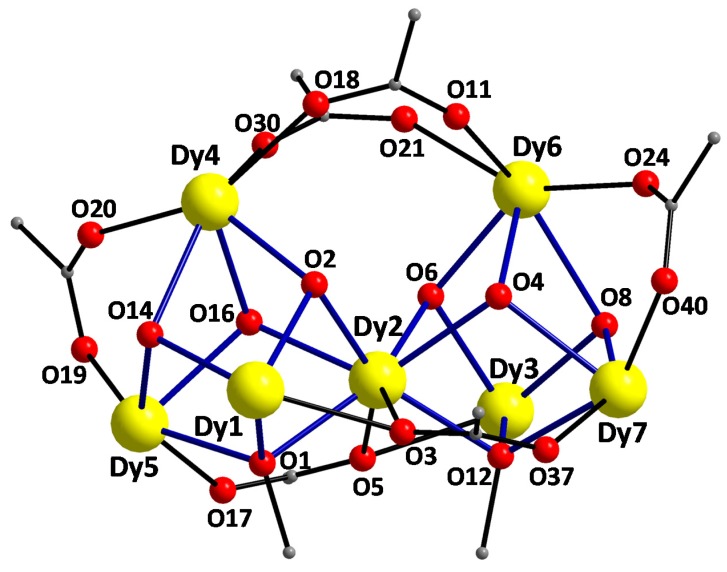
Labelled representation of the complete {Dy_7_(μ_3_-OH)_6_(μ_3_-OMe)_2_(μ_3_-O_2_CR)_2_(μ-O_2_CR)_4_}^7+^ core of **1**, where the RCO_2_^−^-bridging moieties belong to the 2-amino-5-chlorobenzoate part of the nacbH^−^/nacb^2−^ groups. The blue, thick lines highlight the internal dicubane core. Color scheme as in Figure 1.

**Figure 4:**
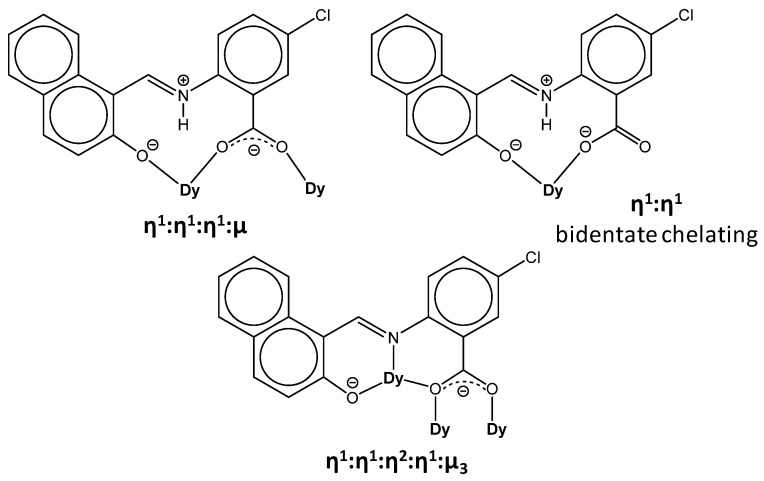
Crystallographically established coordination modes of nacbH^−^ (top) and nacb^2−^ (bottom) ligands present in complex **1**.

The cation of complex **1** (Figure 1) consists of seven Dy^III^ ions held together by six and two μ_3_-bridging OH^−^ and OMe^−^ groups, respectively. This arrangement results in an hourglass-like metal topology, comprising two distorted {Dy_4_(μ_3_-OH)_3_(μ_3_-OMe)}^8+^ cubanes of virtual *C*_2_ symmetry that share a common metal vertex, the Dy2 (Figure 2). Peripheral bridging about the overall {Dy_7_(μ_3_-OH)_6_(μ_3_-OMe)_2_}^13+^ inorganic core (blue thick lines in Figure 3) is provided by the carboxylate moieties of two η^1^:η^1^:η^2^:η^1^:μ_3_ nacb^2−^ and four η^1^:η^1^:η^1^:μ nacbH^−^ ligands (Figure 3 and Figure 4). The remaining two nacbH^−^ groups act as bidentate chelating ligands to Dy1 and Dy3, using their naphthoxido and carboxylate *O*-atoms (Figure 4). The dangling carboxylate *O*-atoms of these nacbH^−^ groups are H-bonded to two μ_3_-OH^−^ ions (O2 and O6, respectively). In all nacbH^−^ groups, there appears to be a migration of the phenol H-atom to the imino *N*-atom; the latter therefore becomes positively charged and—as expected—remains unbound to the metal centers. Finally, additional ligation about the metal core is provided by a monodentate NO_3_^−^ group of 100% occupancy (N9 and its *O*-partners), one bidentate chelating NO_3_^−^ group of 50% occupancy (N13 and its riding *O*-atoms) and two and one terminally bound H_2_O and MeOH solvate molecules, respectively.

**Figure 5:**
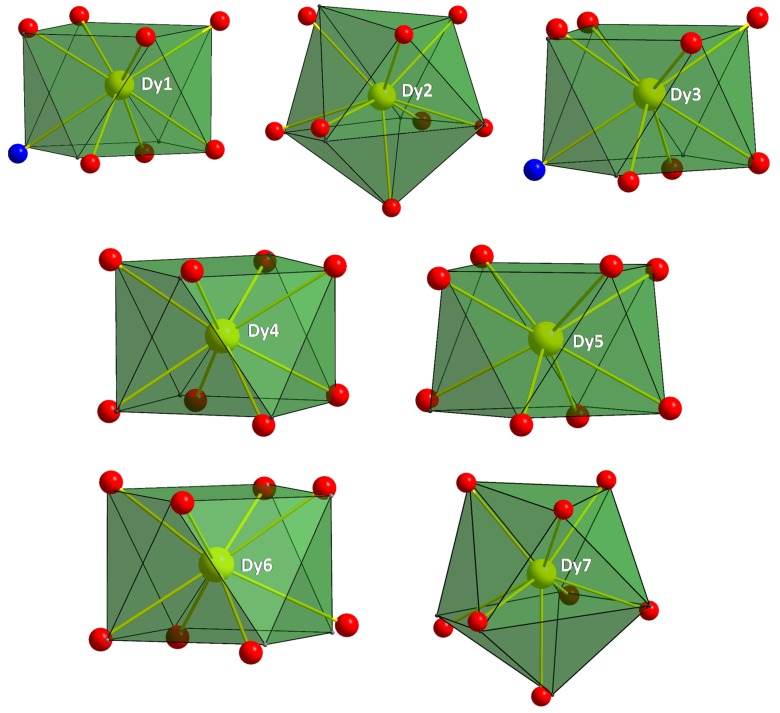
Square antiprismatic and triangular dodecahedral coordination geometries of the Dy atoms in **1**. Points connected by the black, thin lines define the vertices of the ideal polyhedra. Color scheme as in Figure 1.

All Dy^III^ ions in complex **1** are eight-coordinate, and their coordination geometries were determined by the continuous shape measures (CShM) of the SHAPE program [26]. This program is widely used in lanthanide coordination chemistry for the quantitative calculation of the deviation of a specific polyhedron from the ideal shape. The best fits were obtained for the square antiprismatic (CShM values = 1.49, 1.38, 0.84, 0.56 and 0.94 for Dy1, Dy3, Dy4, Dy5 and Dy6, respectively) and triangular dodecahedral (CShM values = 1.52 and 0.86 for Dy2 and Dy7, respectively) geometries (Figure 5). Values of CShM ranging from 0.1 to 3 usually correspond to a small distortion from the ideal geometry [27].

Complex **1** joins a relatively small family of Dy^III^ clusters of nuclearity seven. Since most of these have been reported only within the last several years, we have collected them in Table 2 for a convenient comparison of their structural types and pertinent magnetic data, such as the effective energy barriers (*U*_eff_) for the magnetization reversal (vide infra). Examination of Table 2 reveals that complex **1** has a prototype structural motif, and it displays SMM features with a small *U*_eff_ barrier of similar magnitude with that of the majority of cage-like {Dy_7_} clusters. Exceptions to this rule are only the cyclic-like {Dy_7_} complexes prepared by Collison, Zheng and co-workers.

### 2.3. Solid-State Magnetic Susceptibility Studies

**Figure 6:**
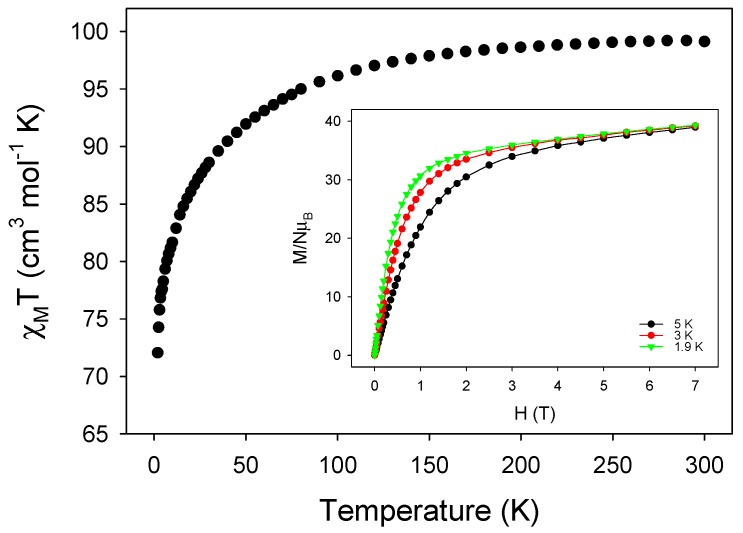
Temperature dependence of the *χ*_Μ_*T* product for **1**∙MeOH at 0.1 T (inset). Plots of magnetization (*M*) versus field (*H*) for **1**∙MeOH at the indicated low temperatures.

Direct current (dc) magnetic susceptibility measurements were performed on a microcrystalline sample of **1**∙MeOH (as analyzed by elemental analyses studies) in the 2–300 K range under an applied magnetic field of 0.1 T (Figure 6). The room temperature *χ*_Μ_*T* value of 99.12 cm^3^ Kmol^−1^ is very close to the theoretical value of 99.19 cm^3^ Kmol^−1^ for seven noninteracting Dy^III^ ions (^6^H_15/2_, *S* = 5/2, *L* = 5 and *g* = 4/3). The *χ*_Μ_*T* product decreases very smoothly on cooling until ~90 K and then, sharply, reaches a value of 72.05 cm^3^ Kmol^−1^ at 2 K. The abrupt decline of the *χ*_Μ_*T* product as the temperature is lowered is mostly due to the depopulation of the crystal field (CF) m*_J_* states and the presence of some weak intramolecular antiferromagnetic interactions between the Dy^III^ ions [37]. This type of magnetic behavior is not uncommon in high-nuclearity Dy^III^ complexes with a closed cage-like topology and a combination of bridging hydroxido, alkoxido and carboxylate groups [38]. The field (*H*) dependence of the magnetization (*M*) at 1.9, 3 and 5 K shows a relatively steep increase at low fields without reaching saturation at 7 T; this behavior is suggestive of the presence of magnetic anisotropy (Figure 6, inset). Moreover, the magnetization value at 7 T is ~36.5 *N*μ_B_, much lower than the expected value of magnetization saturation (*M*_S_) for seven Dy^III^ ions (*M*_S_/*N*μ_B_ = n*g*_J_*J* = 70 *N*μ_B_ for n = 7, *g*_J_ = 4/3 and *J* = 15/2); this response is attributed to the CF effects that induce an appreciable magnetic anisotropy. Furthermore, the reduced magnetization plot of *M/Nμ_B_* versus *H*/*T* (Figure S2) at different magnetic fields (0.1 to 7.0 T) and low temperatures revealed the separation of the isofield curves, in accordance with the presence of magnetic anisotropy and/or low-lying excited *S* states.

**Figure 7:**
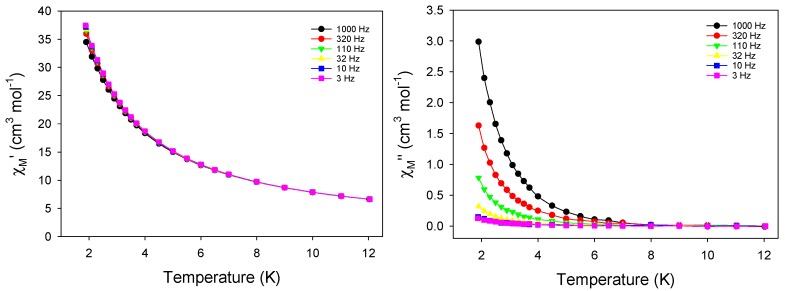
Temperature dependence of the in-phase (*χ*_Μ_′, left) and out-of-phase (*χ*_Μ_′′, right) ac magnetic susceptibilities in a zero dc field for **1**, measured in a 3.0-G ac field oscillating at the frequencies 3–1000 Hz. The solid lines are guides only.

The evaluation of the magnetic dynamics of complex **1** was probed by alternating current (ac) magnetic susceptibility measurements at a zero applied dc field under a weak ac field of 3.0 G oscillating at the frequencies 3–1000 Hz. The compound exhibits frequency-dependent tails of peaks in the in-phase (*χ*_Μ_′) and out-of-phase (*χ*_Μ_′′) susceptibilities versus *T* plots at temperatures below ~8 K, suggesting the onset of slow magnetization relaxation and SMM behavior (Figure 7). The tails of signals are indicative of the presence of the fast quantum tunneling of magnetization (QTM) and, consequently, a small energy barrier for the magnetization reversal. This is a common phenomenon in polynuclear 4f-SMMs with low symmetry structures, in which the metal centers adopt various distorted coordination geometries with a random orientation of their single-ion anisotropies, with respect to the molecular easy-axis. In particular, for Kramers ions, such as Dy^III^, in the majority of coordination environments, the presence of an easy-axis anisotropy, dipole-dipole and hyperfine interactions allow the mixing of the individual Dy^III^ ground states in a zero dc field, thus amplifying the QTM mechanism over the thermally assisted relaxation processes [39]. To overcome the efficient QTM pathway, an external optimum dc field is usually applied to the ac magnetometry, aiming at the shift of the *χ*_Μ_′′ signals at higher temperatures and the observation of entirely visible peaks [20]. However, in the case of complex **1**, a representative diagram of *χ*_Μ_′′ versus dc fields at the lowest possible temperature of 2 K and a fixed ac frequency of 1000 Hz (Figure S3) did not show any peaks of signals at any of the applied dc fields, which suggests that QTM is still the most operative mechanism for magnetization relaxation.

In addition, the Cole-Cole diagrams (Figure S4) for **1** in the temperature range 1.9–8 K did not show any peaks in the semicircular plots, further supporting the presence of fast QTM. No further ac studies were performed under an external dc field, and we have thus focused on quantifying the energy barrier and relaxation time of **1** using the ac data at a zero dc field. Assuming that the magnetization relaxation has only one characteristic time that corresponds to a Debye relaxation process, the SMM parameters can be deduced by applying the Kramers-Kronig equations [40], which result in the combined Equation (2), where *ω* is the angular frequency, *τ*_0_ is the pre-exponential factor, *U*_eff_ is the effective energy barrier for the magnetization reversal and *k_B_* is the Boltzmann’s constant.

ln(*χ*′′/*χ*′) = ln(*ωτ*_0_) + *U*_eff_/*k_B_T* (2)

**Figure 8:**
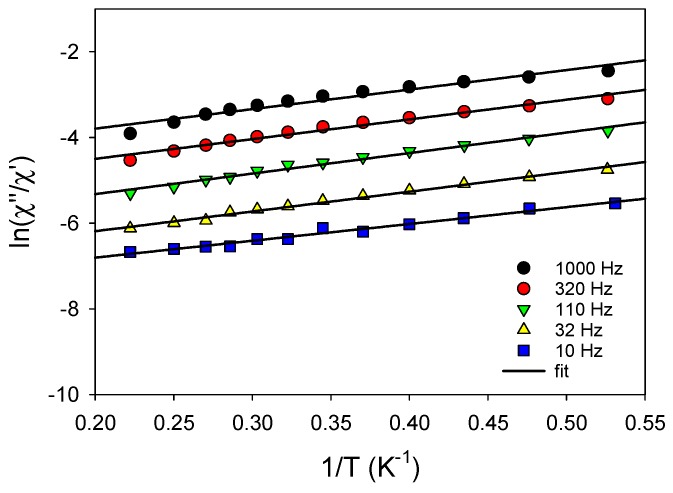
Debye plots of complex **1** for the indicated ac frequencies. The solid lines correspond to the fit of the data by applying Equation (2); see the text for the fit parameters.

Equation (2) is a valuable tool in determining the most important SMM parameters, such as the *U*_eff_ and *τ*_0_, when the out-of-phase peak maxima are not fully resolved in the *χ*′′_M_ versus *T* plots [41]. Based on Equation (2), the best-fit parameters obtained for complex **1** (Figure 8) were: *U*_eff_ = 3.2(1) cm^−1^ (~4.6(1) K) and *τ*_0_ = 3.7(2) × 10^−6^ s, consistent with the expected *τ*_0_ values for a fast-relaxing SMM. The resulting energy barrier is very small, and thus, a thermally assisted Orbach process may be discarded as an operative mechanism for the magnetization relaxation process in complex **1** [42].

## 3. Materials and Methods

### 3.1. Materials, Physical and Spectroscopic Measurements

All manipulations were performed under aerobic conditions using materials (reagent grade) and solvents as received unless otherwise noted. The Schiff base ligand nacbH_2_ was prepared, purified and characterized as described elsewhere [21,22]. Infrared spectra were recorded in the solid state on a Bruker’s FT-IR spectrometer (ALPHA’s Platinum ATR single reflection, in the 4000–400 cm^−1^ range. Elemental analyses (C, H and N) were performed on a Perkin-Elmer 2400 Series II Analyzer. Magnetic susceptibility studies were performed at the temperature range 1.9-300 K using a Quantum Design MPMS XL-7 SQUID magnetometer equipped with a 7 T magnet. Pascal’s constants were used to estimate the diamagnetic correction, which was subtracted from the experimental susceptibility to give the molar paramagnetic susceptibility (χ_M_) [43].

### 3.2. Synthesis of [Dy_7_(OH)_6_(OMe)_2_(NO_3_)_1.5_(nacb)_2_(nacbH)_6_(MeOH)(H_2_O)_2_](NO_3_)_1.5_ (**1**)

To a stirred, yellow solution of nacbH_2_ (0.07 g, 0.20 mmol) and NEt_3_ (84 μL, 0.60 mmol) in a solvent mixture comprising MeOH/MeCN (20 mL, 5:1 *v*/*v*) was added solid Dy(NO_3_)_3_∙5H_2_O (0.09 g, 0.20 mmol). The resulting orange-yellow suspension was stirred for 30 min, during which time, all the solids were dissolved. The solution was then filtered, and the filtrate was left to evaporate slowly at room temperature. After ten days, X-ray quality yellow needle-like crystals of **1**∙5MeOH∙MeCN appeared, and these were collected by filtration, washed with cold MeOH (2 × 2 mL) and MeCN (2 × 2 mL) and dried in the air for 24 h. The yield was 40% (based on the ligand available). The air-dried microcrystalline solid was satisfactorily analyzed as **1**∙MeOH. Anal. calc. for C_148_H_110_N_11_O_45_Dy_7_Cl_8_ (found values in parentheses): C 42.49% (42.61%), H 2.65% (2.76%) and N 3.68% (3.59%). Selected IR data (ATR): *ν* = 1585 (vs), 1539 (s), 1470 (m), 1417 (m), 1352 (s), 1309 (m), 1269 (m), 1211 (m), 1177 (m), 1160 (m), 1141 (m), 1111 (m), 1040 (m), 974 (m), 886 (m), 868 (m), 831 (m), 811 (m), 738 (s), 707 (m), 660 (m), 629 (m), 606 (m), 534 (m), 470 (mb), 438 (m) and 425 (m).

### 3.3. Single-Crystal X-ray Crystallography

A crystal of complex **1** was selected and mounted on MiteGen dual thickness micromounts^TM^ using inert oil. Diffraction data were collected on a D8 VENTURE diffractometer equipped with a multilayer mirror monochromator and a Mo Kα microfocus sealed tube (*λ* = 0.71073 Å). Images were processed with the software SAINT+ [44,45], and absorption effects were corrected with the multi-scan method implemented in SADABS [46]. The structure was solved using the Bruker SHELXTL inside the APEX-III software package and refined using the SHELXLE and PLATON programs [47]. The structure was examined using the Addsym subroutine of PLATON [48] to ensure that no additional symmetry could be applied to the model.

The non-hydrogen atoms of the crystal structure were successfully refined using anisotropic displacement parameters, and the hydrogen atoms bonded to the carbon of the ligands and those of the hydroxyl groups were placed at their idealized positions using the appropriate *HFIX* instructions in SHELXL. All these atoms were included in subsequent refinement cycles in riding-motion approximation with isotropic thermal displacement parameters (*U*_iso_) fixed at 1.2 or 1.5× *U_eq_* of the relative atom. Substantial electron density was found on the data of compound **1**∙5MeOH∙MeCN, most likely due to additional disordered solvate molecules occupying the spaces originated by the close packing of the complex. Our efforts to properly locate, model and refine these residues were unsuccessful, and the investigation for the total potential solvent area using the software package PLATON confirmed clearly the existence of cavities with potential solvent-accessible void volumes. Consequently, the original dataset was treated with the program SQUEEZE [49], a part of the PLATON package of crystallographic software, which calculates the contribution of the smeared electron density in the lattice voids and adds this to the calculated structure factors from the structural model when refining against the .hkl file [50]. All the structural figures of **1** were created using the programs Diamond [51] and Mercury [52].

Unit cell parameters, structure solution and refinement details for **1**∙5MeOH∙MeCN are summarized in Table 3. Further crystallographic details can be found in the corresponding CIF file provided in the ESI. Crystallographic data (excluding structure factors) for the structure reported in this work have been deposited to the Cambridge Crystallographic Data Centre (CCDC) as supplementary publication number: CCDC-1994400. Copies of the data can be obtained online using https://summary.ccdc.cam.ac.uk/structure-summary-form.

## 4. Conclusions and Perspectives

In conclusion, we have herein reported the synthesis, structural and magnetic characterizations of a new heptanuclear Dy^III^ complex **1** with an hourglass-like metal topology, comprising two {Dy_4_(μ_3_-OH)_3_(μ_3_-OMe)}^8+^ cubanes that share a common metal vertex. In addition to the structural interest, complex **1** exhibits slow magnetization relaxation, albeit with a small energy barrier due to the onset of a fast-tunneling mechanism for the magnetization reversal. The employment of the Schiff base ligand *N*-naphthalidene-2-amino-5-chlorobenzoic acid (nacbH_2_) for a first time in 4f-metal chemistry was proved to be a promising route to the preparation of structurally and magnetically appealing molecular nanoscale materials. This work is still in progress, and we are currently trying to alter the chemical, structural and magnetic features of complex **1** by performing chemical reactivity studies, which include variations in the Dy^III^ starting materials, the reaction solvents and the introduction of additional bridging/chelating groups.

## Figures and Tables

**Table 1:** Selected interatomic distances (Å) and angles (°) for complex **1**.

Parameter		Parameter	
Dy1-O34	2.273(6)	Dy4-O30	2.300(6)
Dy1-O13	2.303(6)	Dy4-O18	2.358(6)
Dy1-O14	2.386(6)	Dy4-O16	2.385(6)
Dy1-O3	2.446(6)	Dy4-O14	2.460(6)
Dy1-O10	2.289(6)	Dy5-O25	2.352(6)
Dy1-O2	2.386(6)	Dy5-O1	2.361(6)
Dy1-O1	2.438(6)	Dy5-O19	2.377(6)
Dy1-N8	2.517(8)	Dy5-O33	2.461(6)
Dy2-O2	2.306(6)	Dy5-O16	2.350(6)
Dy2-O1	2.340(6)	Dy5-O17	2.354(6)
Dy2-O4	2.391(6)	Dy5-O14	2.362(6)
Dy2-O5	2.508(5)	Dy5-O32	2.434(6)
Dy2-O6	2.293(6)	Dy6-O11	2.306(6)
Dy2-O12	2.308(6)	Dy6-O29	2.349(6)
Dy2-O16	2.360(6)	Dy6-O24	2.371(6)
Dy2-O3	2.487(6)	Dy6-O8	2.537(6)
Dy3-O35	2.267(6)	Dy6-O6	2.292(5)
Dy3-O27	2.326(6)	Dy6-O28	2.335(7)
Dy3-O8	2.412(6)	Dy6-O21	2.369(6)
Dy3-O5	2.421(6)	Dy6-O4	2.387(6)
Dy3-O22	2.277(6)	Dy7-O37	2.311(7)
Dy3-O12	2.401(6)	Dy7-O39	2.346(6)
Dy3-O6	2.419(6)	Dy7-O40	2.371(6)
Dy3-N7	2.521(7)	Dy7-O38	2.459(7)
Dy4-O2	2.298(6)	Dy7-O8	2.345(6)
Dy4-O31	2.347(6)	Dy7-O4	2.351(6)
Dy4-O15	2.361(6)	Dy7-O12	2.374(5)
Dy4-O20	2.417(6)	Dy7-O41	2.469(7)
Dy5-O1-Dy1	108.7(2)	Dy2-O1-Dy1	94.6(2)
Dy4-O2-Dy1	113.0(2)	Dy4-O2-Dy2	111.7(2)
Dy1-O3-Dy2	90.8(2)	Dy2-O2-Dy1	96.9(2)
Dy7-O4-Dy2	105.1(2)	Dy7-O4-Dy6	104.3(2)
Dy2-O6-Dy6	112.6(2)	Dy6-O4-Dy2	105.9(2)
Dy6-O6-Dy3	113.3(2)	Dy3-O5-Dy2	91.1(2)
Dy7-O8-Dy6	100.0(2)	Dy2-O6-Dy3	96.6(2)
Dy7-O12-Dy3	107.7(2)	Dy7-O8-Dy3	108.3(2)
Dy5-O14-Dy1	110.5(2)	Dy3-O8-Dy6	105.3(2)
Dy1-O14-Dy4	107.4(2)	Dy2-O12-Dy7	107.0(2)
Dy5-O16-Dy2	106.2(2)	Dy2-O12-Dy3	96.7(2)
Dy2-O16-Dy4	106.8(2)	Dy5-O14-Dy4	100.6(2)
Dy2-O1-Dy5	106.5(2)	Dy5-O16-Dy4	103.2(2)

**Table 2:** Structural types and effective energy barriers, *U*_eff_, for heptanuclear Dy^III^ clusters. *^a^*

Complex *^b^*	Type	*U*_eff_	Reference
[Dy_7_(OH)_6_(thmeH_2_)_5_(thmeH)(tpa)_6_(MeCN)_2_]^2+^	metal-centered hexagon	140 K	[28]
[Dy_7_(eddc)(opch)_4_(O_3_PC_10_H_7_)_3_(MeCO_2_)_5_(MeOH)_2_]	ring	74 and 177 K	[29]
[Dy_7_(eddc)_2_(spch)_2_(O_3_PC_10_H_7_)_4_(MeCO_2_)_5_(MeOH)_4_]	ring	45.1 and 48.7 K	[30]
[Dy_7_(OH)_6_(MeOsalox)_2_(MeOsaloxH)_4_(PhCO_2_)_7_(H_2_O)_1.5_(MeOH)_0.5_]	edge-sharing {Dy_3_} triangles	n.r.	[31]
[Dy_7_(L)_6_(OH)_8_(NO_3_)_4_(H_2_O)]^3+^	two incomplete {Dy_3_O_4_} cubes	n.r.	[32]
[Dy_7_(OH)_2_(L’)_9_(aib)]	metal-centered octahedron	n.r.	[33]
[Dy_7_O_2_(OH)_4_(TBC[8]-7*H*)(TBC[8]-6*H*)(DMF)_9_]	capped octahedron	n.r.	[34]
[Dy_7_(OH)_4_(deg)_2_(Hdeg)_2_(O_2_CPh)_11_]	tip-sharing double-butterfly	6 K	[35]
[Dy_7_(OH)_2_(saph)_10_(Me_2_CO)_2_]^−^	two vertex-sharing butterflies	4.3 K	[17]
[Dy_7_(OH)_6_(CO_3_)_3_(sach)_3_(sachH)_3_(MeOH)_6_]	metal-centered trigonal prism	1.7 K	[36]
[Dy_7_(OH)_6_(OMe)_2_(NO_3_)_1.5_(nacb)_2_(nacbH)_6_(MeOH)(H_2_O)_2_]^1.5+^ (**1**)	metal vertex-sharing dicubanes	4.6 K	t.w.

*^a^* Abbreviations: n.r.—not reported; t.w.—this work; thmeH_3—_tris(hydroxymethyl)ethane; tpaH—triphenylacetic acid; eddcH_2_—*N*’,*N*’’-(ethane-1,2-diylidene)-dipyrazine-2-carbohydrazide; opchH_2_—*N*’-(2-hydroxy-3-methoxybenzylidene)pyrazine-2-carbohydrazide; spchH_2_—*N*’-(2-hydroxybenzylidene)pyrazine-2-carbohydrazide; MeOsaloxH_2_—3-methyloxysalicylaldoxime; LH—2-methoxy-6-(pyridin-2-yl-hydrazonomethyl)phenol; aibH—2-aminoisobutyric acid; L′—dianion from the condensation of naphthalene aldehyde and aibH; TBC[8]—*p*-*tert*-butylcalix[8]arene; degH_2_ = diethylene glycol; saphH_2_—*N*-salicylidene-*o*-aminophenol and sachH_2_—*N*-salicylidene-2-aminocyclohexanol. *^b^* Counterions and lattice solvate molecules are omitted.

**Table 3:** Crystallographic data for complex **1**∙5MeOH∙MeCN.

Parameter	1∙5MeOH∙MeCN
Empirical formula	C_154_H_129_N_12_O_49_Dy_7_Cl_8_
FW/g mol^−1^	4352.92
Temperature/K	100(2)
Crystal type	Yellow needle
Crystal size/mm^3^	0.30 × 0.10 × 0.10
Crystal system	Triclinic
Space group	*P*-1
*a*/Å	16.225(1)
*b*/Å	20.227(2)
*c*/Å	28.297(2)
*α*/°	103.56(2)
*β*/°	92.41(2)
*γ*/°	104.18(2)
Volume/Å^3^	8704(1)
*Z*	2
*ρ*_calc/_g cm^−3^	1.641
*μ*/mm^−1^	3.167
F(000)	4188
*θ* range/°	2.17 to 26.02
Radiation	Mo Kα (*λ* = 0.71073)
Index ranges	−20 ≤ *h* ≤ 20 −24 ≤ *k* ≤ 24 −34 ≤ *l* ≤ 34
Reflections collected	307911
Independent reflections	34234 (*R*_int_ = 0.0973)
Goodness-of-fit on *F*^2^	1.052
Final R indexes [*I* ≥ 2σ(*I*)] *^a^*^,*b*^	*R*_1_ = 0.0631 w*R*_2_ = 0.1660
Final R indexes [all data]	*R*_1_ = 0.0878 w*R*_2_ = 0.1788
(Δ*ρ*)_max,min_/e Å^−3^	3.017 and -1.466

*^a^ R*_1_ = Σ(||*F_o_*| − |*F_c_*||)/Σ|*F_o_*|. *^b^* w*R*_2_ = [Σ[*w*(*F*_o_^2^ − *F*_c_^2^)^2^]/Σ[*w*(*F*_o_^2^)^2^]^1/2^, *w* = 1/[*σ* ^2^(*F*_o_^2^) + (*ap*)^2^ + *bp*], where *p* = [max(*F*_o_^2^, 0) + 2*F*_c_^2^]/3.

## Supplementary Materials

The CIF and the checkCIF output files of complex **1**∙5MeOH∙MeCN are available online at https://www.mdpi.com/1420-3049/25/9/2191/s1. In addition, the following figures are available at the same link. Figure S1: A small portion of the crystal packing of complex **1**∙5MeOH∙MeCN, emphasizing with a space-filling model the NO_3_^−^ counterions and the lattice solvate molecules that occupy the voids between the {Dy_7_} clusters. H atoms are omitted for clarity. Figure S2: Plot of reduced magnetization (*M*/*Nμ*_B_) versus *H*/*T* for complex **1** at different fields and temperatures. The solid lines are guides for the eye only. Figure S3: Field (*H*) dependence of the out-of-phase (*χ*_Μ_′′) ac signals for complex **1**, measured at 2.0 K under an ac field of 3.0 G oscillating at a frequency of 1000 Hz. Figure S4: Cole-Cole plots for **1** obtained using the ac susceptibility data at a zero applied dc field.

## References

[1] Atzori M., Sessoli R. (2019). The second quantum revolution: Role and challenges of molecular chemistry. J. Am. Chem. Soc..

[2] Zhang P., Zhang L., Wang C., Xue S., Lin S.-Y., Tang J. (2014). Equatorially coordinated lanthanide single ion magnets. J. Am. Chem. Soc..

[3] Cucinotta G., Perfetti M., Luzon J., Etienne M., Car P.-E., Caneschi A., Calvez G., Bernot K., Sessoli R. (2012). Magnetic anisotropy in a dysprosium/DOTA single-molecule magnet: Beyond simple magneto-structural correlations. Angew. Chem. Int. Ed..

[4] Boulon M.-E., Cucinotta G., Luzon J., Degl’ Innocenti C., Perfetti M., Bernot K., Calvez G., Caneschi A., Sessoli R. (2013). Magnetic anisotropy and spin-parity effect along the series of lanthanide complexes with DOTA. Angew. Chem. Int. Ed..

[5] Woodruff D.N., Winpenny R.E.P., Layfield R.A. (2013). Lanthanide single-molecule magnets. Chem. Rev..

[6] Sessoli R., Powell A.K. (2009). Strategies towards single molecule magnets based on lanthanide ions. Coord. Chem. Rev..

[7] Car P.-E., Perfetti M., Mannini M., Favre A., Caneschi A., Sessoli R. (2011). Giant field dependence of the low temperature relaxation of the magnetization in a dysprosium(III)-DOTA complex. Chem. Commun..

[8] Lucaccini E., Sorace L., Perfetti M., Costes J.-P., Sessoli R. (2014). Beyond the anisotropy barrier: Slow relaxation of the magnetization in both easy-axis and easy-plane Ln(trensal) complexes. Chem. Commun..

[9] Gatteschi D., Sessoli R., Villain J. (2006). Molecular Nanomagnets.

[10] Bagai R., Christou G. (2009). The *Drosophila* of single-molecule magnetism: [Mn_12_O_12_(O_2_CR)_16_(H_2_O)_4_]. Chem. Soc. Rev..

[11] Rinehart J.D., Long J.R. (2011). Exploiting single-ion anisotropy in the design of f-element single-molecule magnets. Chem. Sci..

[12] Guo F.-S., Day B.M., Chen Y.-C., Tong M.-L., Mansikkamäki A., Layfield R.A. (2018). Magnetic hysteresis up to 80 kelvin in a dysprosium metallocene single-molecule magnet. Science.

[13] Layfield R., Murugesu M. (2015). Lanthanides and Actinides in Molecular Magnetism.

[14] Zheng X.-Y., Xie J., Kong X.-J., Long L.-S., Zheng L.-S. (2019). Recent advances in the assembly of high-nuclearity lanthanide clusters. Coord. Chem. Rev..

[15] Zhang Z., Zhang Y., Zheng Z., Zheng Z. (2016). Lanthanide hydroxide cluster complexes via ligand-controlled hydrolysis of the lanthanide ions. Recent Development in Clusters of Rare Earths and Actinides: Chemistry and Materials; Struct. Bonding.

[16] Alexandropoulos D.I., Nguyen T.N., Cunha-Silva L., Zafiropoulos T.F., Escuer A., Christou G., Stamatatos T.C. (2013). Slow magnetization relaxation in unprecedented Mn^III^_4_Dy^III^_3_ and Mn^III^_4_Dy^III^_5_ clusters from the use of *N*-salicylidene-*o*-aminophenol. Inorg. Chem..

[17] Mazarakioti E.C., Cunha-Silva L., Bekiari V., Escuer A., Stamatatos T.C. (2015). New structural topologies in 4f-metal cluster chemistry from vertex-sharing butterfly units: {Ln^III^_7_} complexes exhibiting slow magnetization relaxation and ligand-centred emissions. RSC Adv..

[18] Athanasopoulou A.A., Pilkington M., Raptopoulou C.P., Escuer A., Stamatatos T.C. (2014). Structural aesthetics in molecular nanoscience: A unique Ni_26_ cluster with a ‘rabbit-face’ topology and a discrete Ni_18_ ‘molecular chain’. Chem. Commun..

[19] Pantelis K.N., Karotsis G., Lampropoulos C., Cunha-Silva L., Escuer A., Stamatatos T.C. (2020). ‘Metal complexes as ligands’ for the synthesis of coordination polymers: A Mn_III_ monomer as a building block for the preparation of an unprecedented 1-D {Mn^II^Mn^III^}_n_ linear chain. Materials.

[20] Mazarakioti E.C., Regier J., Cunha-Silva L., Wernsdorfer W., Pilkington M., Tang J., Stamatatos T.C. (2017). Large energy barrier and magnetization hysteresis at 5 K for a symmetric {Dy_2_} complex with spherical tricapped trigonal prismatic Dy^III^ ions. Inorg. Chem..

[21] Perlepe P.S., Cunha-Silva L., Bekiari V., Gagnon K.J., Teat S.J., Escuer A., Stamatatos T.C. (2016). Structural diversity in Ni^II^ cluster chemistry: Ni_5_, Ni_6_, and {NiNa_2_}_n_ complexes bearing the Schiff-base ligand *N*-naphthalidene-2-amino-5-chlorobenzoic acid. Dalton Trans..

[22] Perlepe P.S., Cunha-Silva L., Gagnon K.J., Teat S.J., Lampropoulos C., Escuer A., Stamatatos T.C. (2016). “Ligands-with-benefits”: Naphthalene-substituted Schiff bases yielding new Ni^II^ metal clusters with ferromagnetic and emissive properties and undergoing exciting transformations. Inorg. Chem..

[23] Papatriantafyllopoulou C., Moushi E.E., Christou G., Tasiopoulos A.J. (2016). Filling the gap between the quantum and classical worlds of nanoscale magnetism: Giant molecular aggregates based on paramagnetic 3d metal ions. Chem. Soc. Rev..

[24] Li X.-Y., Su H.-F., Li Q.-W., Feng R., Bai H.-Y., Chen H.-Y., Xu J., Bu X.-H. (2019). A giant Dy_76_ cluster: A fused bi-nanopillar structural model for lanthanide clusters. Angew. Chem. Int. Ed..

[25] Liu W., Thorp H.H. (1993). Bond valence sum analysis of metal-ligand bond lengths in metalloenzymes and model complexes. 2. Refined distances and other enzymes. Inorg. Chem..

[26] Llunell M., Casanova D., Girera J., Alemany P., Alvarez S. (2010). SHAPE.

[27] Alvarez S., Alemany P., Casanova D., Cirera J., Llunell M., Avnir D. (2005). Shape maps and polyhedral interconversion paths in transition metal chemistry. Coord. Chem. Rev..

[28] Sharples J.W., Zheng Y.-Z., Tuna F., McInnes E.J.L., Collison D. (2011). Lanthanide discs chill well and relax slowly. Chem. Commun..

[29] Tian H., Bao S.-S., Zheng L.-M. (2016). Cyclic single-molecule magnets: From the odd-numbered heptanuclear to a dimer of heptanuclear dysprosium clusters. Chem. Commun..

[30] Tian H., Bao S.-S., Zheng L.-M. (2016). Cyclic single-molecule magnets: From even-numbered hexanuclear to odd-numbered heptanuclear dysprosium clusters. Eur. J. Inorg. Chem..

[31] Guo F.-S., Guo P.-H., Meng Z.-S., Tong M.-L. (2011). Slow magnetic relaxation in a novel heptanuclear Dy_7_ cluster with five edge-sharing Dy_3_ triangles. Polyhedron.

[32] Goura J., Walsh J.P.S., Tuna F., Chandrasekhar V. (2015). Synthesis, structure, and magnetism of non-planar heptanuclear lanthanide(III) complexes. Dalton Trans..

[33] Canaj A.B., Tzimopoulos D.I., Philippidis A., Kostakis G.E., Milios C.J. (2012). A strongly blue-emitting heptametallic [Dy^III^_7_] centered-octahedral single-molecule magnet. Inorg. Chem..

[34] Taylor S.M., Sanz S., McIntosh R.D., Beavers C.M., Teat S.J., Brechin E.K., Dalgarno S.J. (2012). *p-tert*-butylcalix[8]arene: An extremely versatile platform for cluster formation. Chem. Eur. J..

[35] Peng G., Zhang Y.-Y., Li Z.-Y., Kostakis G.E. (2017). First examples of polynuclear lanthanide diethylene glycol based coordination clusters. Eur. J. Inorg. Chem..

[36] Mazarakioti E.C., Poole K.M., Cunha-Silva L., Christou G., Stamatatos T.C. (2014). A new family of Ln_7_ clusters with an ideal D_3h_ metal-centered trigonal prismatic geometry, and SMM and photoluminescence behaviors. Dalton Trans..

[37] Tang J., Zhang P. (2015). Lanthanide Single Molecule Magnets.

[38] Peng J.B., Kong X.J., Zhang Q.C., Orendáč M., Prokleška J., Ren Y.P., Long L.S., Zheng Z., Zheng L.S. (2014). Beauty, symmetry, and magnetocaloric effect—Four-shell keplerates with 104 lanthanide atoms. J. Am. Chem. Soc..

[39] Liddle S.T., Van Slageren J. (2015). Improving f-element single molecule magnets. Chem. Soc. Rev..

[40] Cole K.S., Cole R.H. (1941). Dispersion and absorption in dielectrics I. Alternating current characteristics. J. Chem. Phys..

[41] Bartolomé J., Filoti G., Kuncser V., Schinteie G., Mereacre V., Anson C.E., Powell A.K., Prodius D., Turta C. (2009). Magnetostructural correlations in the tetranuclear series of {Fe_3_LnO_2_} butterfly core clusters: Magnetic and Mössbauer spectroscopic study. Phys. Rev. B..

[42] Guo Y.-N., Xu G.-F., Guo Y., Tang J. (2011). Relaxation dynamics of dysprosium(III) single molecule magnets. Dalton Trans..

[43] Bain G.A., Berry J.F. (2008). Diamagnetic corrections and Pascal’s constants. J. Chem. Educ..

[44] (2005). SAINT+, Data Integration Engine.

[45] (2006). APEX2, Data Collection Software.

[46] Sheldrick G.M. (1998). SADABS v.2.01, Bruker/Siemens Area Detector Absorption Correction Program.

[47] Hübschle C.B., Sheldrick G.M., Dittrich B. (2011). ShelXle a Qt graphical user interface for SHELXL. J. Appl. Cryst..

[48] Spek A.L. (2009). Structure validation in chemical crystallography. Acta Crystallogr. D.

[49] Spek A.L. (2003). Single-crystal structure validation with the program PLATON. J. Appl. Crystallogr..

[50] Spek A.L. (1990). PLATON, An integrated tool for the analysis of the results of a single crystal structure determination. Acta Cryst. A.

[51] Bradenburg K. (2008). DIAMOND, Visual Crystal Structure Information System.

[52] Bruno I.J., Cole J.C., Edgington P.R., Kessler M.K., Macrae C.F., McCabe P., Pearson J., Taylor R. (2002). New software for searching the Cambridge structural database and visualizing crystal structures. Acta Cryst..

