# Supplementary material for: 4f-Metal Clusters Exhibiting Slow Relaxation of Magnetization: A {Dy7} Complex with An Hourglass-like Metal Topology
Source: Molecules. 2020 May 7;25(9):2191. doi: 10.3390/molecules25092191 (PMC7249001; doi:10.3390/molecules25092191)

checkCIF/PLATON report

You have not supplied any structure factors. As a result the full set of tests cannot be run.

THIS REPORT IS FOR GUIDANCE ONLY. IF USED AS PART OF A REVIEW PROCEDURE FOR PUBLICATION, IT SHOULD NOT REPLACE THE EXPERTISE OF AN EXPERIENCED CRYSTALLOGRAPHIC REFEREE.

No syntax errors found. CIF dictionary Interpreting this report

Datablock: LK15

```
Bond precision:  C-C = 0.0155 A          Wavelength=0.71073

Cell:           a=16.2248(14)      b=20.2271(17)    c=28.297(2)
                alpha=103.560(2)   beta=92.408(2)   gamma=104.177(2)
Temperature:    100 K
```

	Calculated	Reported
Volume	8703.9(12)	8703.8(13)
Space group	P -1	P -1
Hall group	-P 1	-P 1
Moiety formula	C147 H101.50 Cl8 Dy7 N10 O42.50, N O3, C2 H3 N, 2.75(C H4 O)	?
Sum formula	C151.75 H115.50 Cl8 Dy7 N12 O48.25	C3.99 H3.04 Cl0.21 Dy0.18 N0.32 O1.27
Mr	4300.17	113.16
Dx,g cm-3	1.641	1.641
Z	2	76
Mu (mm-1)	3.167	3.167
F000	4188.0	4188.0
F000’	4189.53	
h,k,lmax	20,24,34	20,24,34
Nref	34264	34234
Tmin,Tmax	0.691,0.729	0.450,0.742
Tmin’	0.383	

```
Correction method= # Reported T Limits: Tmin=0.450 Tmax=0.742
AbsCorr = ?

Data completeness= 0.999          Theta(max)= 26.022

R(reflections)= 0.0631( 25380)    wR2(reflections)= 0.1788( 34234)

S = 1.052                   Npar= 2079
```

The following ALERTS were generated. Each ALERT has the format
test-name_ALERT_alert-type_alert-level.
Click on the hyperlinks for more details of the test.

Alert level A

```
SHFSU01_ALERT_2_A  The absolute value of parameter shift to su ratio > 0.20
            Absolute value of the parameter shift to su ratio given   6.902
            Additional refinement cycles may be required.
```

Author Response: Multiple disorders and significant residual electron density around th

```
PLAT080_ALERT_2_A Maximum Shift/Error ...........................       6.90 Why ?
```

Author Response: Multiple disorders and significant residual electron density around th

```
PLAT417_ALERT_2_A Short Inter D-H..H-D       H32A     ..H53      .       0.97 Ang.
                                                      x,y,z  =      1_555 Check
```

Author Response: Multiple disorders and significant residual electron density around th

```
PLAT602_ALERT_2_A VERY LARGE Solvent Accessible VOID(S) in Structure        ! Info
```

Author Response: Multiple solvent disorders - SQUEEZE (part of PLATON was used.

Alert level B

```
PLAT201_ALERT_2_B Isotropic non-H Atoms in Main Residue(s) .......          3 Report
              O42     O43     C109
PLAT220_ALERT_2_B NonSolvent Resd 1  C   Ueq(max) / Ueq(min) Range        7.0 Ratio
PLAT420_ALERT_2_B D-H Without Acceptor       O39      --H39B     .     Please Check
PLAT420_ALERT_2_B D-H Without Acceptor       O47      --H47'     .     Please Check
PLAT420_ALERT_2_B D-H Without Acceptor       O54      --H54      .     Please Check
```

Alert level C

```
ABSMU01_ALERT_1_C  The ratio of given/expected absorption coefficient lies
               outside the range 0.99 <> 1.01
            Calculated value of mu =     3.100
            Value of mu given      =     3.167
PLAT041_ALERT_1_C Calc. and Reported SumFormula    Strings  Differ     Please Check
PLAT052_ALERT_1_C Info on Absorption Correction Method   Not Given     Please Do !
PLAT094_ALERT_2_C Ratio of Maximum / Minimum Residual Density ....       2.06 Report
PLAT213_ALERT_2_C Atom Cl1              has ADP max/min Ratio .....        3.1 prolat
PLAT215_ALERT_3_C Disordered O44        has ADP max/min Ratio .....        3.2 Note
PLAT215_ALERT_3_C Disordered N13        has ADP max/min Ratio .....        3.3 Note
PLAT220_ALERT_2_C NonSolvent Resd 1  O   Ueq(max) / Ueq(min) Range        5.5 Ratio
PLAT222_ALERT_3_C NonSolvent Resd 1  H   Uiso(max)/Uiso(min) Range        7.9 Ratio
PLAT242_ALERT_2_C Low    MainMol Ueq as Compared to Neighbors of        O12 Check
PLAT260_ALERT_2_C Large Average Ueq of Residue Including       O45      0.134 Check
PLAT329_ALERT_4_C Carbon Atom Hybridisation Unclear for ..........       C109 Check
PLAT342_ALERT_3_C Low Bond Precision on  C-C Bonds ...............    0.01548 Ang.
PLAT369_ALERT_2_C Long   C(sp2)-C(sp2) Bond  C124     - C130     .       1.53 Ang.
PLAT369_ALERT_2_C Long   C(sp2)-C(sp2) Bond  C131     - C132     .       1.53 Ang.
```

```
PLAT410_ALERT_2_C Short Intra H...H Contact  H80      ..H83      .       1.99 Ang.
                                                      x,y,z  =      1_555 Check
PLAT410_ALERT_2_C Short Intra H...H Contact  H119     ..H122     .       1.94 Ang.
                                                      x,y,z  =      1_555 Check
```

Alert level G

```
CELLZ01_ALERT_1_G Difference between formula and atom_site contents detected.
CELLZ01_ALERT_1_G ALERT: Large difference may be due to a
            symmetry error - see SYMMG tests
           From the CIF: _cell_formula_units_Z   76
           From the CIF: _chemical_formula_sum  C3.99 H3.04 Cl0.21 Dy0.18 N0.32 O1
           TEST: Compare cell contents of formula and atom_site data

           atom    Z*formula  cif sites diff
           C        303.24    303.50   -0.26
           H        231.04    231.00    0.04
           Cl        15.96     16.00   -0.04
           Dy        13.68     14.00   -0.32
           N         24.32     24.00    0.32
           O         96.52     96.50    0.02
PLAT002_ALERT_2_G Number of Distance or Angle Restraints on AtSite          8 Note
PLAT003_ALERT_2_G Number of Uiso or Uij Restrained non-H Atoms ...        218 Report
PLAT007_ALERT_5_G Number of Unrefined Donor-H Atoms ..............         19 Report
PLAT012_ALERT_1_G N.O.K.   _shelx_res_checksum Found in CIF ......     Please Check
PLAT013_ALERT_1_G N.O.K.   _shelx_hkl_checksum Found in CIF ......     Please Check
PLAT045_ALERT_1_G Calculated and Reported Z Differ by a Factor ...       0.03 Check
PLAT083_ALERT_2_G SHELXL Second Parameter in WGHT  Unusually Large      79.47 Why ?
PLAT154_ALERT_1_G The s.u.'s on the Cell Angles are Equal ..(Note)      0.002 Degree
PLAT172_ALERT_4_G The CIF-Embedded .res File Contains DFIX Records          1 Report
PLAT177_ALERT_4_G The CIF-Embedded .res File Contains DELU Records          1 Report
PLAT187_ALERT_4_G The CIF-Embedded .res File Contains RIGU Records          1 Report
PLAT300_ALERT_4_G Atom Site Occupancy of O44        Constrained at        0.5 Check
PLAT300_ALERT_4_G Atom Site Occupancy of N10        Constrained at        0.5 Check
PLAT300_ALERT_4_G Atom Site Occupancy of N13        Constrained at        0.5 Check
PLAT300_ALERT_4_G Atom Site Occupancy of H10A       Constrained at        0.5 Check
PLAT300_ALERT_4_G Atom Site Occupancy of H10B       Constrained at        0.5 Check
PLAT300_ALERT_4_G Atom Site Occupancy of H10C       Constrained at        0.5 Check
PLAT300_ALERT_4_G Atom Site Occupancy of O45        Constrained at        0.5 Check
PLAT300_ALERT_4_G Atom Site Occupancy of O55        Constrained at        0.5 Check
PLAT300_ALERT_4_G Atom Site Occupancy of O56        Constrained at        0.5 Check
PLAT300_ALERT_4_G Atom Site Occupancy of N14        Constrained at        0.5 Check
PLAT300_ALERT_4_G Atom Site Occupancy of O50        Constrained at        0.5 Check
PLAT300_ALERT_4_G Atom Site Occupancy of O51        Constrained at        0.5 Check
PLAT300_ALERT_4_G Atom Site Occupancy of O52        Constrained at        0.5 Check
PLAT300_ALERT_4_G Atom Site Occupancy of N12        Constrained at        0.5 Check
PLAT300_ALERT_4_G Atom Site Occupancy of O47        Constrained at        0.5 Check
PLAT300_ALERT_4_G Atom Site Occupancy of C2'        Constrained at        0.5 Check
PLAT300_ALERT_4_G Atom Site Occupancy of H15G       Constrained at        0.5 Check
PLAT300_ALERT_4_G Atom Site Occupancy of H15H       Constrained at        0.5 Check
PLAT300_ALERT_4_G Atom Site Occupancy of H15I       Constrained at        0.5 Check
PLAT300_ALERT_4_G Atom Site Occupancy of H47'       Constrained at        0.5 Check
PLAT300_ALERT_4_G Atom Site Occupancy of O49        Constrained at        0.5 Check
PLAT300_ALERT_4_G Atom Site Occupancy of C154       Constrained at        0.5 Check
PLAT300_ALERT_4_G Atom Site Occupancy of H15M       Constrained at        0.5 Check
PLAT300_ALERT_4_G Atom Site Occupancy of H15N       Constrained at        0.5 Check
PLAT300_ALERT_4_G Atom Site Occupancy of H15O       Constrained at        0.5 Check
PLAT300_ALERT_4_G Atom Site Occupancy of H49'       Constrained at        0.5 Check
PLAT300_ALERT_4_G Atom Site Occupancy of O54        Constrained at        0.5 Check
PLAT300_ALERT_4_G Atom Site Occupancy of C1'        Constrained at        0.5 Check
PLAT300_ALERT_4_G Atom Site Occupancy of H1'1       Constrained at        0.5 Check
```

```
PLAT300_ALERT_4_G Atom Site Occupancy of H1’2       Constrained at        0.5 Check
PLAT300_ALERT_4_G Atom Site Occupancy of H1’3       Constrained at        0.5 Check
PLAT300_ALERT_4_G Atom Site Occupancy of H54        Constrained at        0.5 Check
PLAT300_ALERT_4_G Atom Site Occupancy of O46        Constrained at       0.25 Check
PLAT300_ALERT_4_G Atom Site Occupancy of C129       Constrained at       0.25 Check
PLAT300_ALERT_4_G Atom Site Occupancy of H12A       Constrained at       0.25 Check
PLAT300_ALERT_4_G Atom Site Occupancy of H12B       Constrained at       0.25 Check
PLAT300_ALERT_4_G Atom Site Occupancy of H12C       Constrained at       0.25 Check
PLAT300_ALERT_4_G Atom Site Occupancy of H46        Constrained at       0.25 Check
PLAT301_ALERT_3_G Main Residue  Disorder .............(Resd  1  )          1% Note
PLAT302_ALERT_4_G Anion/Solvent/Minor-Residue Disorder (Resd  2  )       100% Note
PLAT302_ALERT_4_G Anion/Solvent/Minor-Residue Disorder (Resd  3  )       100% Note
PLAT302_ALERT_4_G Anion/Solvent/Minor-Residue Disorder (Resd  5  )       100% Note
PLAT302_ALERT_4_G Anion/Solvent/Minor-Residue Disorder (Resd  6  )       100% Note
PLAT302_ALERT_4_G Anion/Solvent/Minor-Residue Disorder (Resd  8  )       100% Note
PLAT302_ALERT_4_G Anion/Solvent/Minor-Residue Disorder (Resd  9  )       100% Note
PLAT304_ALERT_4_G Non-Integer Number of Atoms in ..... (Resd  9  )       1.50 Check
PLAT333_ALERT_2_G Large Aver C6-Ring C-C Dist C1       -C10      .       1.42 Ang.
PLAT333_ALERT_2_G Large Aver C6-Ring C-C Dist C112     -C121     .       1.43 Ang.
PLAT412_ALERT_2_G Short Intra XH3 .. XHn     H01C     ..H10A     .       1.67 Ang.
                                                     x,y,z  =      1_555 Check
PLAT413_ALERT_2_G Short Inter XH3 .. XHn     H15O     ..H69      .       1.77 Ang.
                                                   x,1+y,z  =      1_565 Check
PLAT413_ALERT_2_G Short Inter XH3 .. XHn     H1’3     ..H41      .       2.11 Ang.
                                                   1+x,y,z  =      1_655 Check
PLAT414_ALERT_2_G Short Intra D-H..H-X       H10B     ..H39A             1.98 Ang.
                                                     x,y,z  =      1_555 Check
PLAT417_ALERT_2_G Short Inter D-H..H-D       H25B     ..H49’     .       0.89 Ang.
                                                     x,y,z  =      1_555 Check
```

Author Response: Multiple disorders and significant residual electron density around th

```
PLAT431_ALERT_2_G Short Inter HL..A Contact  Cl4      ..O42      .       3.09 Ang.
                                                1-x,-y,1-z  =      2_656 Check
PLAT432_ALERT_2_G Short Inter X...Y Contact  Cl6      ..C2’              3.22 Ang.
                                               1-x,1-y,2-z  =      2_667 Check
PLAT432_ALERT_2_G Short Inter X...Y Contact  C42      ..C92              3.19 Ang.
                                                  -1+x,y,z  =      1_455 Check
PLAT720_ALERT_4_G Number of Unusual/Non-Standard Labels ..........          8 Note
PLAT721_ALERT_1_G Bond    Calc     0.98000, Rep     1.11360 Dev...       0.13 Ang.
              C1’      -H1’1           1.555   1.555 ........     #   13 Check
PLAT721_ALERT_1_G Bond    Calc     0.97000, Rep     1.10930 Dev...       0.14 Ang.
              C1’      -H1’2           1.555   1.555 ........     #   14 Check
PLAT721_ALERT_1_G Bond    Calc     0.99000, Rep     1.11780 Dev...       0.13 Ang.
              C1’      -H1’3           1.555   1.555 ........     #   15 Check
PLAT721_ALERT_1_G Bond    Calc     0.94000, Rep     0.97350 Dev...       0.03 Ang.
              O46      -H46            1.555   1.555 ........     #  131 Check
PLAT721_ALERT_1_G Bond    Calc     0.95000, Rep     0.96050 Dev...       0.01 Ang.
              O54      -H54            1.555   1.555 ........     #  142 Check
PLAT721_ALERT_1_G Bond    Calc     0.98000, Rep     1.00560 Dev...       0.03 Ang.
              C109     -H10A           1.555   1.555 ........     #  342 Check
PLAT721_ALERT_1_G Bond    Calc     0.98000, Rep     1.00600 Dev...       0.03 Ang.
              C109     -H10B           1.555   1.555 ........     #  343 Check
PLAT721_ALERT_1_G Bond    Calc     0.98000, Rep     1.00600 Dev...       0.03 Ang.
              C109     -H10C           1.555   1.555 ........     #  344 Check
PLAT721_ALERT_1_G Bond    Calc     0.98000, Rep     0.99780 Dev...       0.02 Ang.
              C129     -H12A           1.555   1.555 ........     #  377 Check
PLAT721_ALERT_1_G Bond    Calc     0.99000, Rep     1.00690 Dev...       0.02 Ang.
              C129     -H12B           1.555   1.555 ........     #  378 Check
PLAT721_ALERT_1_G Bond    Calc     0.97000, Rep     1.00390 Dev...       0.03 Ang.
```

```
              C129     -H12C            1.555   1.555 ........     #  379 Check
PLAT722_ALERT_1_G Angle   Calc      109.00, Rep      123.50 Dev...      14.50 Degree
              O54     -C1'     -H1'1       1.555  1.555  1.555     #   56 Check
PLAT722_ALERT_1_G Angle   Calc      110.00, Rep      124.10 Dev...      14.10 Degree
              O54     -C1'     -H1'2       1.555  1.555  1.555     #   57 Check
PLAT722_ALERT_1_G Angle   Calc      110.00, Rep       92.30 Dev...      17.70 Degree
              H1'1    -C1'     -H1'2       1.555  1.555  1.555     #   58 Check
PLAT722_ALERT_1_G Angle   Calc      109.00, Rep      124.20 Dev...      15.20 Degree
              O54     -C1'     -H1'3       1.555  1.555  1.555     #   59 Check
PLAT722_ALERT_1_G Angle   Calc      109.00, Rep       91.80 Dev...      17.20 Degree
              H1'1    -C1'     -H1'3       1.555  1.555  1.555     #   60 Check
PLAT722_ALERT_1_G Angle   Calc      110.00, Rep       91.50 Dev...      18.50 Degree
              H1'2    -C1'     -H1'3       1.555  1.555  1.555     #   61 Check
PLAT722_ALERT_1_G Angle   Calc      109.00, Rep       54.50 Dev...      54.50 Degree
              DY6     -O4      -H4'        1.555  1.555  1.555     #  436 Check
PLAT722_ALERT_1_G Angle   Calc      111.00, Rep       51.80 Dev...      59.20 Degree
              DY2     -O4      -H4'        1.555  1.555  1.555     #  437 Check
PLAT722_ALERT_1_G Angle   Calc      113.00, Rep       44.90 Dev...      68.10 Degree
              DY3     -O8      -H8''       1.555  1.555  1.555     #  448 Check
PLAT722_ALERT_1_G Angle   Calc      108.00, Rep       60.90 Dev...      47.10 Degree
              DY6     -O8      -H8''       1.555  1.555  1.555     #  449 Check
PLAT722_ALERT_1_G Angle   Calc      121.00, Rep      117.50 Dev...       3.50 Degree
              C129    -O46     -H46        1.555  1.555  1.555     #  502 Check
PLAT722_ALERT_1_G Angle   Calc      119.00, Rep      120.60 Dev...       1.60 Degree
              C154    -O49     -H49'       1.555  1.555  1.555     #  505 Check
PLAT722_ALERT_1_G Angle   Calc      108.00, Rep       20.60 Dev...      87.40 Degree
              C48     -N2      -H2'        1.555  1.555  1.555     #  513 Check
PLAT722_ALERT_1_G Angle   Calc      112.00, Rep        8.30 Dev...     103.70 Degree
              C61     -N5      -H5'        1.555  1.555  1.555     #  522 Check
PLAT722_ALERT_1_G Angle   Calc      112.00, Rep        8.70 Dev...     103.30 Degree
              C79     -N6      -H6'        1.555  1.555  1.555     #  525 Check
PLAT722_ALERT_1_G Angle   Calc      109.00, Rep      111.80 Dev...       2.80 Degree
              O12     -C109    -H10A       1.555  1.555  1.555     #  874 Check
PLAT722_ALERT_1_G Angle   Calc      109.00, Rep      112.00 Dev...       3.00 Degree
              O12     -C109    -H10B       1.555  1.555  1.555     #  875 Check
PLAT722_ALERT_1_G Angle   Calc      109.00, Rep      106.80 Dev...       2.20 Degree
              H10A    -C109    -H10B       1.555  1.555  1.555     #  876 Check
PLAT722_ALERT_1_G Angle   Calc      110.00, Rep      112.00 Dev...       2.00 Degree
              O12     -C109    -H10C       1.555  1.555  1.555     #  877 Check
PLAT722_ALERT_1_G Angle   Calc      109.00, Rep      106.90 Dev...       2.10 Degree
              H10A    -C109    -H10C       1.555  1.555  1.555     #  878 Check
PLAT722_ALERT_1_G Angle   Calc      110.00, Rep      106.90 Dev...       3.10 Degree
              H10B    -C109    -H10C       1.555  1.555  1.555     #  879 Check
PLAT722_ALERT_1_G Angle   Calc      109.00, Rep      113.70 Dev...       4.70 Degree
              O46     -C129    -H12B       1.555  1.555  1.555     #  938 Check
PLAT722_ALERT_1_G Angle   Calc      109.00, Rep      107.80 Dev...       1.20 Degree
              H12A    -C129    -H12B       1.555  1.555  1.555     #  939 Check
PLAT722_ALERT_1_G Angle   Calc      110.00, Rep      112.40 Dev...       2.40 Degree
              O46     -C129    -H12C       1.555  1.555  1.555     #  940 Check
PLAT722_ALERT_1_G Angle   Calc      110.00, Rep      106.70 Dev...       3.30 Degree
              H12A    -C129    -H12C       1.555  1.555  1.555     #  941 Check
PLAT722_ALERT_1_G Angle   Calc      110.00, Rep      106.50 Dev...       3.50 Degree
              H12B    -C129    -H12C       1.555  1.555  1.555     #  942 Check
PLAT779_ALERT_4_G Suspect or Irrelevant (Bond) Angle(s) in CIF . #        448 Check
              DY3  -O8   -H8''    1.555   1.555   1.555            44.90 Deg.
PLAT779_ALERT_4_G Suspect or Irrelevant (Bond) Angle(s) in CIF . #        513 Check
              C48  -N2   -H2'     1.555   1.555   1.555            20.60 Deg.
PLAT779_ALERT_4_G Suspect or Irrelevant (Bond) Angle(s) in CIF . #        522 Check
              C61  -N5   -H5'     1.555   1.555   1.555             8.30 Deg.
PLAT779_ALERT_4_G Suspect or Irrelevant (Bond) Angle(s) in CIF . #        525 Check
              C79  -N6   -H6'     1.555   1.555   1.555             8.70 Deg.
PLAT790_ALERT_4_G Centre of Gravity not Within Unit Cell: Resd.  #          8 Note
```

```
              C H4 O
PLAT794_ALERT_5_G Tentative Bond Valency for Dy4       (III)     .       3.01 Info
PLAT794_ALERT_5_G Tentative Bond Valency for Dy5       (III)     .       2.88 Info
PLAT794_ALERT_5_G Tentative Bond Valency for Dy6       (III)     .       3.02 Info
PLAT802_ALERT_4_G CIF Input Record(s) with more than 80 Characters          3 Info
PLAT860_ALERT_3_G Number of Least-Squares Restraints .............       3396 Note
PLAT883_ALERT_1_G No Info/Value for _atom_sites_solution_primary .     Please Do !
```

```
   4 ALERT level A = Most likely a serious problem - resolve or explain
   5 ALERT level B = A potentially serious problem, consider carefully
  17 ALERT level C = Check. Ensure it is not caused by an omission or oversight
 118 ALERT level G = General information/check it is not something unexpected

  47 ALERT type 1 CIF construction/syntax error, inconsistent or missing data
  31 ALERT type 2 Indicator that the structure model may be wrong or deficient
   6 ALERT type 3 Indicator that the structure quality may be low
  56 ALERT type 4 Improvement, methodology, query or suggestion
   4 ALERT type 5 Informative message, check
```

It is advisable to attempt to resolve as many as possible of the alerts in all categories. Often the minor alerts point to easily fixed oversights, errors and omissions in your CIF or refinement strategy, so attention to these fine details can be worthwhile. In order to resolve some of the more serious problems it may be necessary to carry out additional measurements or structure refinements. However, the purpose of your study may justify the reported deviations and the more serious of these should normally be commented upon in the discussion or experimental section of a paper or in the "special_details" fields of the CIF. checkCIF was carefully designed to identify outliers and unusual parameters, but every test has its limitations and alerts that are not important in a particular case may appear. Conversely, the absence of alerts does not guarantee there are no aspects of the results needing attention. It is up to the individual to critically assess their own results and, if necessary, seek expert advice.

Publication of your CIF in IUCr journals

A basic structural check has been run on your CIF. These basic checks will be run on all CIFs submitted for publication in IUCr journals (*Acta Crystallographica*, *Journal of Applied Crystallography*, *Journal of Synchrotron Radiation*); however, if you intend to submit to *Acta Crystallographica Section C* or *E* or *IUCrData*, you should make sure that full publication checks are run on the final version of your CIF prior to submission.

Publication of your CIF in other journals

Please refer to the *Notes for Authors* of the relevant journal for any special instructions relating to CIF submission.

PLATON version of 22/12/2019; check.def file version of 13/12/2019

Datablock LK15 - ellipsoid plot

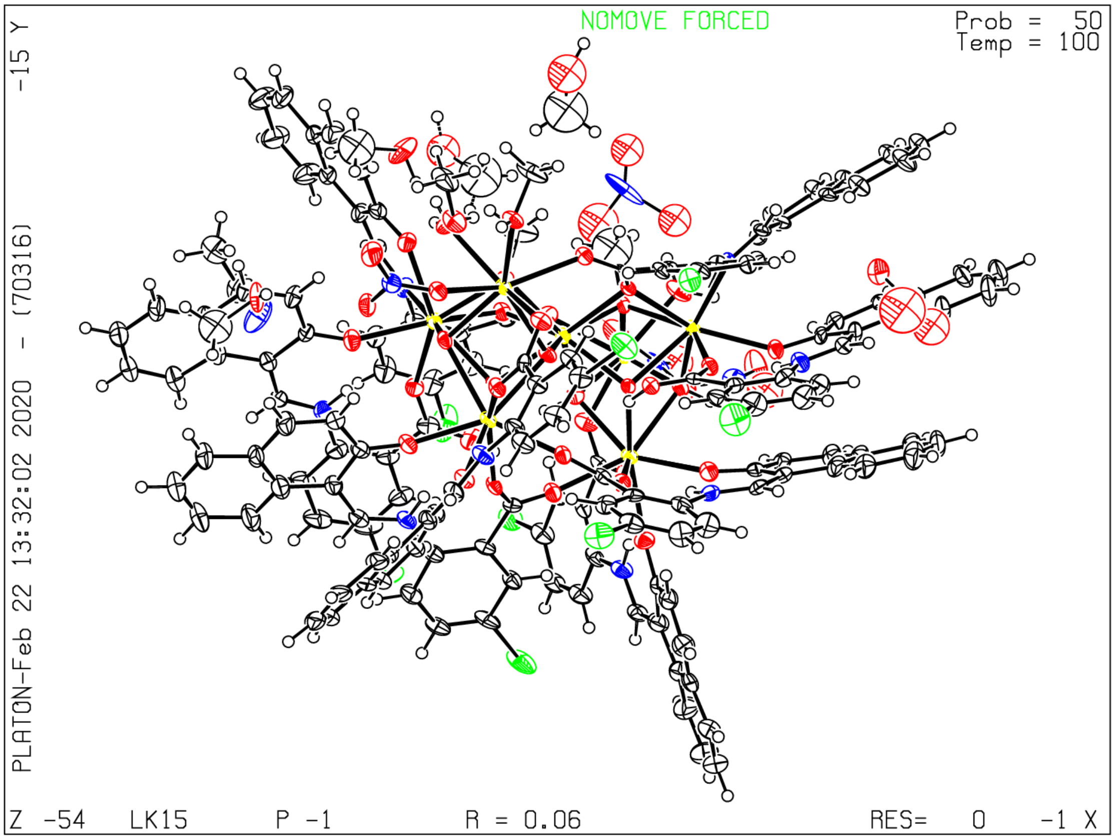

Supplement: Supplementary file 1 [file molecules-25-02191-s001.zip › checkcif.pdf]
