# Supplementary material for: 4f-Metal Clusters Exhibiting Slow Relaxation of Magnetization: A {Dy7} Complex with An Hourglass-like Metal Topology
Source: Molecules. 2020 May 7;25(9):2191. doi: 10.3390/molecules25092191 (PMC7249001; doi:10.3390/molecules25092191)
Supplement: Supplementary file 1 [file molecules-25-02191-s001.zip › Dy7 nacb paper_Stamatatos v1_ESI_R1.pdf]

molecules

Electronic Supplementary Information

4f-Metal Clusters Exhibiting Slow Relaxation of Magnetization: A {Dy_7} Complex with an Hourglass-like Metal Topology

Konstantinos N. Pantelis [1], Panagiota S. Perlepe [1], Spyridon Grammatikopoulos [1], Christos Lampropoulos [2], Jinkui Tang [3] and Theocharis C. Stamatatos [1,*]

[1] Chemistry Department, University of Patras, Patras 265 04, Greece; kostaspantelis95@gmail.com; pennyperlepes@gmail.com; spiridongramma@upatras.gr

[2] Department of Chemistry, University of North Florida, 1 UNF Dr., Jacksonville, FL 32224, USA; christosl1981@gmail.com

[3] State Key Laboratory of Rare Earth Resource Utilization, Changchun Institute of Applied Chemistry, Chinese Academy of Sciences, Changchun 130022, P. R. China; tang@ciac.ac.cn

* Correspondence: thstama@upatras.gr; Tel.: +30-2610996008

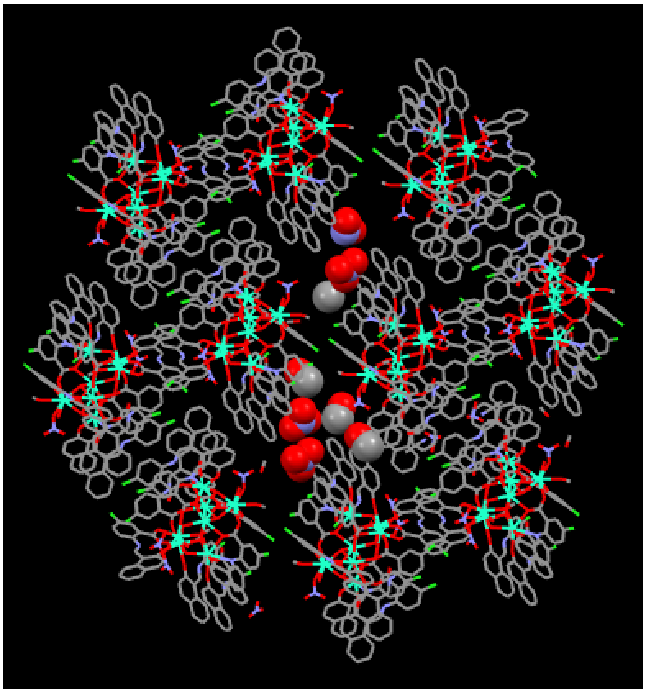

Figure S1. A small portion of the crystal packing of complex **1**·5MeOH·MeCN, emphasizing with a space-filling model the NO_3^- counterions and the lattice solvate molecules that occupy the voids between the {Dy_7} clusters. H atoms are omitted for clarity.

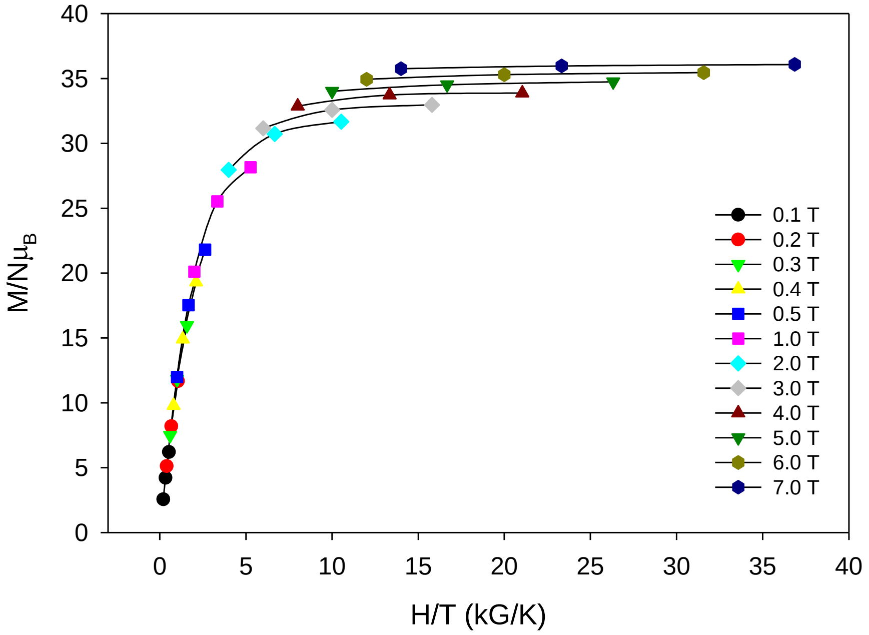

Figure S2. Plot of reduced magnetization ($M/N\mu_B$) *vs* H/T for complex **1** at different fields and temperatures. The solid lines are guides for the eye only.

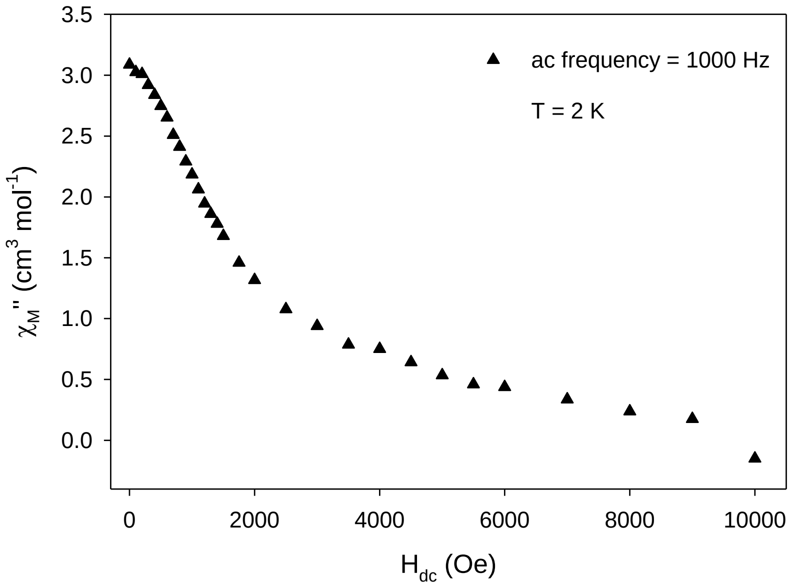

Figure S3. Field (H) dependence of the out-of-phase (χ_M'') ac signals for complex **1**, measured at 2.0 K under an ac field of 3.0 G oscillating at a frequency of 1000 Hz.

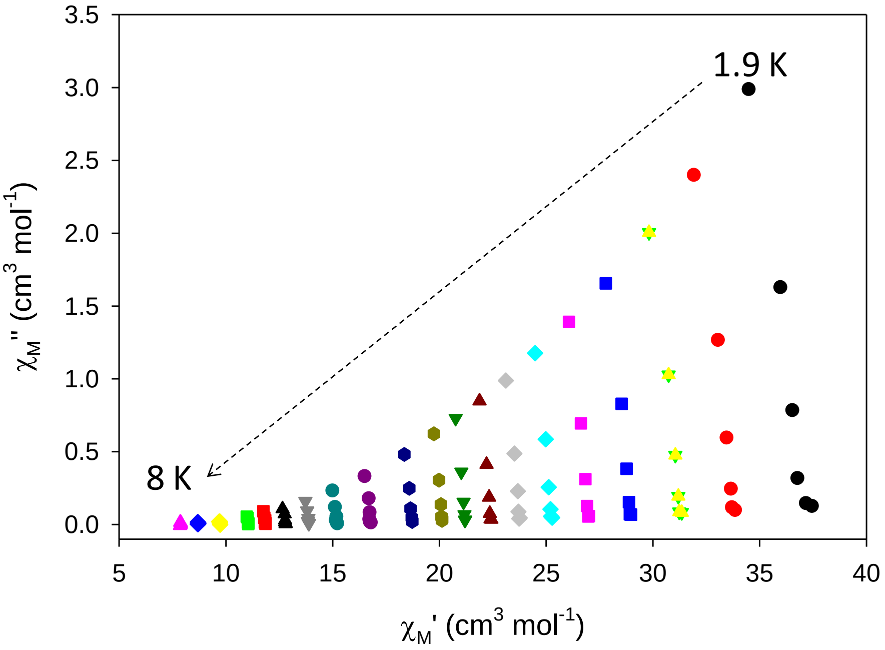

Figure S4. Cole-Cole plots for **1** obtained using the ac susceptibility data at zero applied dc field.
